# Proteomic Analysis of Proton Beam Irradiated Human Melanoma Cells

**DOI:** 10.1371/journal.pone.0084621

**Published:** 2014-01-02

**Authors:** Sylwia Kedracka-Krok, Urszula Jankowska, Martyna Elas, Urszula Sowa, Jan Swakon, Agnieszka Cierniak, Pawel Olko, Bozena Romanowska-Dixon, Krystyna Urbanska

**Affiliations:** 1 Faculty of Biochemistry, Biophysics and Biotechnology, Jagiellonian University, Kraków, Poland; 2 Institute of Nuclear Physics, PAS, Kraków, Poland; 3 Department of Ophthalmology and Ophthalmic Oncology, Jagiellonian University Medical College, Kraków, Poland; 4 Malopolska Centre of Biotechnology, Krakow, Poland; University of Tennessee, United States of America

## Abstract

Proton beam irradiation is a form of advanced radiotherapy providing superior distributions of a low LET radiation dose relative to that of photon therapy for the treatment of cancer. Even though this clinical treatment has been developing for several decades, the proton radiobiology critical to the optimization of proton radiotherapy is far from being understood. Proteomic changes were analyzed in human melanoma cells treated with a sublethal dose (3 Gy) of proton beam irradiation. The results were compared with untreated cells. Two-dimensional electrophoresis was performed with mass spectrometry to identify the proteins. At the dose of 3 Gy a minimal slowdown in proliferation rate was seen, as well as some DNA damage. After allowing time for damage repair, the proteomic analysis was performed. In total 17 protein levels were found to significantly (more than 1.5 times) change: 4 downregulated and 13 upregulated. Functionally, they represent four categories: (i) DNA repair and RNA regulation (VCP, MVP, STRAP, FAB-2, Lamine A/C, GAPDH), (ii) cell survival and stress response (STRAP, MCM7, Annexin 7, MVP, Caprin-1, PDCD6, VCP, HSP70), (iii) cell metabolism (TIM, GAPDH, VCP), and (iv) cytoskeleton and motility (Moesin, Actinin 4, FAB-2, Vimentin, Annexin 7, Lamine A/C, Lamine B). A substantial decrease (2.3 x) was seen in the level of vimentin, a marker of epithelial to mesenchymal transition and the metastatic properties of melanoma.

## Introduction

Proton therapy is used worldwide to treat several types of cancer due to superior targeting and energy deposition [1], [2]. Uveal melanoma is especially well suited for this kind of radiotherapy, as precise dose delivery is crucial to maintain eye function. In spite of the fact that proton therapy is used clinically with great success, not much is known about the biological effects of proton radiation. A substantial body of data has been accumulated on the biological effectiveness of proton radiation [3]–[13], and on some mechanisms of proton-induced cell death [14]–[17], but many other biological effects of the proton beam are unclear [1]. As proton irradiation is considered to be low-LET radiation (<20 keV/µm), its biological effects are assumed to be similar to those induced by photon radiation. However, there is some experimental data demonstrating that this is not always the case [1], [18].

As a stressor and a factor in cell death, proton radiation, as with other types of radiation, induces DNA damage, followed by DNA repair and a cellular stress response cascade. However, in comparison with photon radiation, proton therapy has been observed to be more effective against photon-radioresistant cell lines [4], [7], [12], [19] and different DNA repair responses were triggered, e.g. no ATR (ataxia-telangiectasia and Rad3-related protein kinases) activation being observed [20]. In terms of DNA damage, there was a prevalence of oxidative base damage [14], more double strand breaks [21]–[23] and larger DNA repair foci [24], [25]. Apoptosis was induced at doses above 10 Gy [14], [17] and cell cycle arrest in the G2-M phase was observed [11]. Interestingly, a differential oxidative stress response gene expression was recorded [16], [18].

Several cellular signaling cascades were induced by proton irradiation. For example, in PC3 cells irradiated with 10 Gy, it induced the cell cycle checkpoint ataxia-telangiectasia, mutated gene (ATM) and its downstream targets, such as p53, p21, and bax-α (Bcl-2–associat GTPase KRas protein and cyclin F, in contrast to gamma radiation which upregulates cell cycle blockers, such as Cyclin-dependent kinase inhibitor 2A [26]. p38, c-Jun N-terminal kinases (JNK) and Mitogen-activated protein kinases (MAP) signaling turned out to be crucial for proton irradiation–induced apoptosis, whereas pro-survival ERK (Extracellular Signal-regulated Kinase) activation, although typical for gamma-radiation, was absent after proton irradiation [1]. Other differences were also found in the regulation of angiogenesis, metastasis and migration properties, and inflammation [1].

The goal of this study was to characterize the cellular response to a sublethal dose of proton beam irradiation in a comprehensive way at protein level. Proteomic analysis was performed using BLM cells irradiated with 3 Gy of a 60 keV proton beam, passaged for 28–35 days to allow cellular repair. Our hypothesis was that a low dose of proton beam irradiation affects the proteins implicated in DNA repair, cellular stress response and survival even as a delayed outcome of proton beam irradiation. A significant (more than 1.5 × change) upregulation of 13 proteins and a downregulation of 4 proteins was found.

## Materials and Methods

### Cells

Human melanoma BLM is a highly metastasizing cell line. It was derived from a lung metastasis of the human melanoma Bro subline implanted in a nude mouse as earlier described [27], [28] and was cultured in an RPMI medium with 10% fetal bovine serum and antibiotics. The BLM cells were a kind gift from Prof. Martine J. Jager from Leiden University.

### Proton Beam Irradiation

Cells in suspension in phosphate buffer saline (PBS) at 1×10^6^ cells/ml were transported on ice to the proton beam facility. 1.5 ml of the cell suspension was irradiated at RT in an Eppendorf vial placed in a positioning holder. The source of the 58 MeV proton beam was the AIC-144 cyclotron at The Institute of Nuclear Physics, Polish Academy of Sciences, Kraków. The cells were irradiated with the dose of 3.0 Gy (CGE). The dose rate was 0.15 Gy/s. Irradiated cells were transferred to the medium and plated at 1×10^5^ cell/ml in 10 cm Petri dishes. After 7 passages of irradiated BLM cells (4–5 weeks in culture after irradiation), they were isolated and were used for proteomic analysis. Non-irradiated BLM cells served as the control.

### Cell Growth and Comet Assay

For the proliferation assay, 1,000 or 5,000 cells were seeded in each well of a 96-well plate. At selected time points cells were removed by trypsin, stained with trypan blue and the number of living cells was counted using a Burker cytometer. The level of DNA damage was determined by the electrophoresis of single cells in agarose gel as earlier described [29], [30]. Briefly, the cell suspension was mixed with low melting point agarose, set on slides, lysed and neutralized in appropriate buffers. Electrophoresis was performed at 23 V (0.74 V/cm, 300 mA) for 30 min at 4°C. All stages of the experiment were carried out in the dark to eliminate any extra DNA damages. Prior to analysis the slides were stained with propidium iodide (2.5 µg/ml). The analysis of DNA damage was carried out with COMET PLUS 2.9 software (Comet Plus, Theta System Gmbh, Germany). The percentage content of DNA in the comet’s tail (TDC) was determined from 100 random images of comets per slide. The analysis was done in two replicates.

### Proteomic Analysis

#### Sample preparation

The cells were washed two times in 1 ml of a wash solution (10 mM tris(hydroxymethyl)-aminomethane (Tris), 5 mM magnesium acetate, pH 8.0) and centrifuged at 400 g for 10 min at 20°C. The cell pellet was lysed in an ice-cold buffer that contained 7 M urea, 2 M thiourea, 4% 3-[(3- cholamidopropyl) dimethylammonio] -1-propanesulfonate (CHAPS), 40 mM Tris pH 8.5, 65 mM Dithiothreitol (DTT), 1 mM EDTA and 1 mM phenylmethylsulphonyl fluoride. Cell disruption was achieved by sonication at 320 W, 20 kHz, 30 s ON/30 s OFF, two times for 10 minutes with Bioruptor UCD-200 (Diogenode, Liege, Belgium). The cell lysate was centrifuged at 15 000 g for 30 min at 12°C to remove debris. Proteins were precipitated using methanol and chloroform according to Wessel and Flügge [31]. The protein pellet was solubilized in a rehydration buffer that was composed of 7 M urea, 2 M thiourea, 2% CHAPS, 0.002% bromophenol blue, 0.5% immobilized pH gradient (IPG) buffer and 20 mM DTT (the IPG buffer and DTT were added just before use). Protein concentration was measured by Bradford assay [32].

#### Two-dimensional electrophoresis

Isoelectric focusing (IEF) was carried out with an Ettan IPGphor 3 IEF System (GE Healthcare, Uppsala, Sweden). 24-cm strips with an immobilized pH 3–10 nonlinear gradient (GE Healthcare) were passively rehydrated for 16 h with 450 µl rehydration buffer containing 200 µg of total protein. The IEF was performed at 50 µA/strip for 80 000 Vh at 20°C. Prior to Sodium dodecyl sulfate - Polyacrylamide gel electrophoresis the IPG strips were equilibrated using a two-step procedure, each being gently shaken for 10 min in a 75 mM Tris–HCl pH 8.8 buffer that contained 30% glycerol, 6 M urea and 2% SDS. In the first step, the proteins were reduced by the addition of 1% DTT to the buffer. In the second, they were alkylated with 2.5% iodoacetamide. After IEF the proteins were separated on 12.5% polyacrylamide gels (255 · 296 · 1 · mm). The gels were cast using an Ettan DALTsix Gel Caster (GE Healthcare). Electrophoresis was performed on an Ettan DALTsix Large Vertical System (GE Healthcare) at 2 W/gel for 45 min and 16 W/gel for 3.5 h at 20°C. After electrophoresis, the gels were fixed overnight in 50% methanol, 3% phosphoric acid and stained with Colloidal Coomassie Blue according to the following protocol. The gels were washed 3×30 min with water. In the first step of staining, the gels were incubated in a 400 ml solution consisting of 34% methanol, 3% phosphoric acid and 17% ammonium sulfate. After 1 hour, 140 mg Coomassie Blue G-250 was added to each gel and incubated for the next 5 days. Before scanning on ImageScanner III (GE Healthcare, Uppsala, Sweden) the gels were washed 3×30 minutes with water. The gel images were analyzed by ImageMaster 2D Platinum v 7.0 (GE Healthcare).

#### Protein identification by mass spectrometry

To identify the content of the protein spots of interest, the gel pieces were manually excised, destained at 37°C by washing several times in 25% and 50% acetonitrile in a 25 mM ammonium bicarbonate buffer (NH_4_HCO_3_). The gel pieces were dehydrated in 100% acetonitrile (ACN) and dried in a Speedvac for 5 min, then 15 *µ*L trypsin (Biocentrum) solution (10 ng/*µ*L in 25 mM NH_4_HCO_3_, pH 8.0) was added and incubated for 15 min. After that an additional 20 *µ*L of 25 mM NH_4_HCO_3_ was added. Digestion was carried out overnight at 37°C. Peptides were extracted by sonication and drying with 100% ACN. The extracts were evaporated to dryness and resuspended in 2% ACN with 0,05% TFA. Peptides were analyzed using the UltiMate 3000RS LCnanoSystem (Dionex) coupled with a MicrOTOF-QII mass spectrometer (Bruker) using nano online ESIsprayer.

To briefly describe the of peptide analysis, the peptides were injected on a C18 precolumn (Acclaim PepMap Nano trap Column) using 2% ACN with 0,05% TFA as the mobile phase. They were further separated on a 15 cm×75 µm RP column (Acclaim PepMap 75 µm 100 Å Nano Series TM Column) using gradient 2–40% ACN in 0.05% FA for 30 minutes. MS was operated in standard DDA (data dependent acqusition) MS/MS mode with fragmentation of the most intensive precursor ions.

The MS spectra measured were recalibrated using the fragment ions of trypsin derived peptides. The Mascot Generic format (.mgf) was generated by pre-processing the raw data with Data Analysis 4.0 software (Bruker, Germany). The resulting lists of peaks were used to search the non-redundant protein database Swissprot with the taxonomic restriction – Homo sapiens (20 321 sequences) using an in-house Mascot server (v.2.3.0, Matrix Science, London, UK). The following search parameters were applied: enzyme specificity – trypsin, permitted number of missed cleavages –1, fixed modification – carbamidomethylation (C), variable modifications – oxidation (M), phosphorylation (STY), methylation (DE), protein mass – unrestricted, peptide mass tolerance – ±20 ppm, fragment mass tolerance – ±0.05 Da.

## Results

### Cell Growth Inhibition and DNA Damage

**Figure 1:**
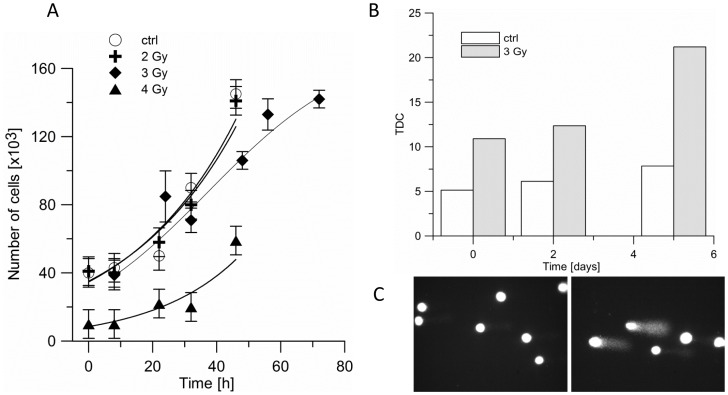
The effect of proton beam irradiation on BLM melanoma cells. A. Proliferation of BLM cells after proton beam irradiation with doses 0 (open circle), 2 (cross), 3 (diamond) and 4 Gy (triangle). Cells were irradiated in suspension, and then plated in 96-well plates. B, C. DNA damage in untreated (white bar), and irradiated with 3 Gy of proton beam (stripped bar) BLM cells are presented in terms of the percentage of DNA that left the comet's head and was found in the comet’s tail after electrophoresis (TDC).

3 Gy of proton beam irradiation slowed down cell growth in culture by app. 15% (Fig. 1 A). Starting from day 4, the number of viable cells in culture decreased. This is in agreement with the increase in DNA damage shortly after the treatment (Fig. 1B,C). The cells were kept in culture for several passages (4–5 weeks) before the proteomic analysis was performed, in order to allow for cellular repair.

### Proteomic Analysis

**Figure 2:**
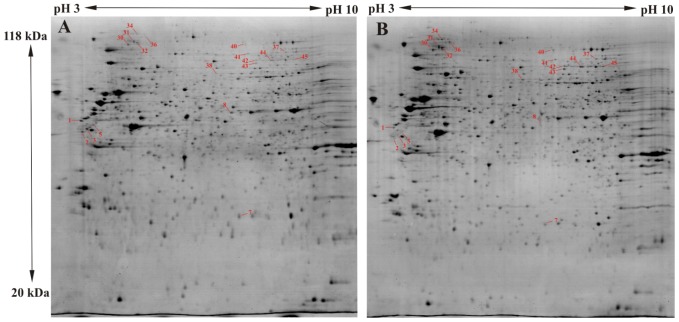
Representative 2D electrophoresis maps stained with Colloidal Commassie. The results obtained for each experimental group are shown: control BLM cells (untreated) (**A**), 3 Gy proton beam irradiated BLM cells (**B**).

**Figure 3:**
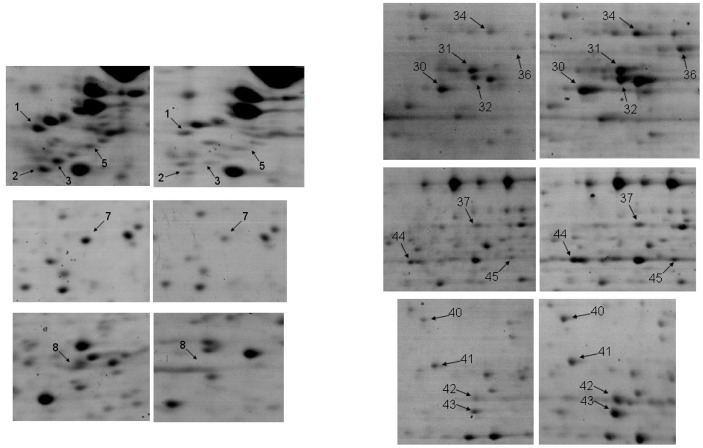
The zoomed changes in the level of spots identified by MS in the two groups. Left panel – control group, Right panel –3 Gy irradiated group. Spot numbers as listed in Table 1.

The mean number of identified spots per gel was 1200 and the average number of matches per gel was 860 (71%). 22 differential spots were found which fulfilled the Student t-test (Table 1). A representative spot pattern is depicted in Fig. 2. The protein contents of all differential spots were identified by LC-MS/MS on the basis of peptide mass matching following digestion with trypsin, and their peptide sequences which were obtained in a fragmentation process. The SwissProt accession numbers, the abbreviated and full names of the proteins, their theoretical pI and MW values are presented in Table 1. It also shows data from the mass spectrometry analysis, such as their score and the protein amino acid sequence coverage by matching peptides and the number of unique peptides. For the majority the identification was very reliable with a very high score. Only in the case of two spots were two proteins identified where it was not possible to specify the source of the difference. Finally, 17 regulated proteins were identified, 4 of them being downregulated, and 13 upregulated in the proton irradiated samples in comparison to the control group. The protein which showed the most pronounced downregulation after proton irradiation was vimentin, which was found in four spots. Detailed analysis of the MS/MS spectra for vimentin revealed differences in the methylation pattern of the protein isoforms. In spot number 1 methylation was recognized at 7 glutamic acid residues (109, 134, 136, 151, 151, 153, 225), in spots no. 2 and 3 only at glutamic acid in position 151, while in spot number 5 no modification was found. Although methylation typically occurs at lysine and arginine, there are also reports of the important biological role of the methylation of glutamic acid [33], [34]. In Fig. 3 the differential spots are shown.

**Table 1:** List of differentially regulated proteins from a comparison of proton beam irradiated and unirradiated BLM cells.

Spot no^a^	Accession nr^b^	Protein	MW [kDa] calculated	pI	MW [kDa]in gel	Scores	#Peptides	SC^c^ [%]	Ratio^d^ ir/ctrl	fold change	p-value
**1**	**VIME_HUMAN**	**Vimentin**	**53.6**	**4.9**	**52.6**	**2736.8**	**41**	**60.3**	**0.49**	**−2.0**	0.018
**2**	**VIME_HUMAN**	**Vimentin**	**53.6**	**4.9**	**48.7**	**1866.5**	**29**	**51.9**	**0.46**	**−2.2**	0.031
**3**	**VIME_HUMAN**	**Vimentin**	**53.6**	**4.9**	**49.7**	**2230.9**	**36**	**65.5**	**0.29**	**−3.4**	0.001
**5**	**VIME_HUMAN**	**Vimentin**	**53.6**	**4.9**	**51.1**	**781.7**	**15**	**36.9**	**0.64**	**−1.6**	0.015
**7**	**TPIS_HUMAN**	**Triosephosphate isomerase**	**30.8**	**5.6**	**31.4**	**889.2**	**10**	**52.1**	**0.46**	**−2.2**	0.017
**8**	**ANXA7_HUMAN**	**Annexin A7**	**52.7**	**5.4**	**55.0**	**637.2**	**8**	**15.0**	**0.40**	**−2.5**	0.032
**46**	**G3P_HUMAN**	**Glyceraldehyde-3-phosphate dehydrogenase**	**36.0**	**9.3**	**35.0**	**932.7**	**47**	**24.0**	**0.42**	**−2.4**	0.003
**30**	**TERA_HUMAN**	**Transitional endoplasmic reticulum ATPase**	**89.3**	**5.0**	**104.3**	**3308.2**	**46**	**59.3**	**1.89**	**1.9**	0.015
**31**	**LMNB2_HUMAN**	**Lamin-B2**	**67.6**	**5.2**	**108.8**	**2304.7**	**35**	**48.3**	**1.38**	**1.4**	0.005
**32**	**ACTN4_HUMAN**	**Alpha-actinin-4**	**104.8**	**5.2**	**106.7**	**4204.7**	**61**	**69.9**	**1.88**	**1.9**	0.043
**34**	**CAPR1_HUMAN**	**Caprin-1**	**78.3**	**5.0**	**117.1**	**192.5**	**3**	**4.8**	**1.78**	**1.8**	0.038
**36**	**MVP_HUMAN**	**Major vault protein**	**99.3**	**5.2**	**112.6**	**1937.7**	**29**	**43.9**	**1.95**	**1.9**	0.012
**37**	**FUBP2_HUMAN**	**Far upstream element-binding protein 2**	**73.1**	**7.0**	**94.6**	**1464.6**	**26**	**32.1**	**1.72**	**1.7**	0.042
**38**	ACTN1_HUMAN	Alpha-actinin-1	103.0	5.1	75.5	2786.0	43	59.2	**1.85**	**1.8**	0.049
	LMNA_HUMAN	Prelamin-A/C	74.1	6.6		2069.0	32	42.9			
**40**	**PDC6I_HUMAN**	**Programmed cell death 6-interacting protein**	**96.0**	**6.1**	**104.1**	**1739.9**	**25**	**37.9**	**1.55**	**1.6**	0.029
**41**	**MCM7_HUMAN**	**DNA replication licensing factor MCM7**	**81.3**	**6.1**	**94.1**	**1574.3**	**25**	**37.0**	**1.66**	**1.7**	0.047
**42**	**LMNA_HUMAN**	**Prelamin-A/C**	**74.1**	**6.6**	**87.1**	**2285.0**	**30**	**47.0**	**1.59**	**1.6**	0.028
**43**	**MOES_HUMAN**	**Moesin**	**67.8**	**6.0**	**84.5**	**1807.4**	**29**	**42.6**	**2.36**	**2.4**	0.013
**44**	**LMNA_HUMAN**	**Prelamin-A/C**	**74.1**	**6.6**	**86.6**	**1881.7**	**23**	**37.3**	**2.45**	**2.5**	0.027
**45**	**LMNA_HUMAN**	**Prelamin-A/C**	**74.1**	**6.6**	**87.0**	**2508.5**	**33**	**50.8**	**1.46**	**1.5**	0.033
**47**	HSP71_HUMAN	Heat shock 70 kDa protein 1	70.0	5.4	64.5	1611.1	61	35.0	**1.81**	**1.8**	0.082
	G3BP1_HUMAN	Ras GTPase-activating protein-bindingprotein 1	52.1	5.3		969.4	55	24.0			
**48**	**STRAP_HUMAN**	**Serine-threonine kinase receptor-associated protein**	**38.4**	**4.8**	**42.0**	**866.1**	**58**	**15.0**	**4.16**	**4.2**	0.006

Spots were compared by 2D stained with Colloidal Coomassie and proteins were identified using LC-MS/MS.

^a^ The spot location is shown in Figure 2 ad 3.

^b^ Protein accession number from the UniProtKB/Swiss-Prot nonredundant protein database.

^c^ obtained sequence coverage.

^d^ Ratio calculated in relation to unirradiated control group.

## Discussion

Our initial hypothesis was that a sublethal dose of proton beam irradiation would upregulate the proteins implicated in DNA repair, inflammation, stress response and the regulation of survival and, that it would possibly downregulate those connected to the metabolism, or to protein production. However, the data revealed a slightly different picture. First of all, relatively few protein levels were changed after irradiation in comparison with ionizing radiation [35], [36]. Moreover, relatively few proteins engaged in DNA repair were upregulated. These unexpected results may arise from the long period given for recuperation after the irradiation insult. Another unanticipated result is the lack of inflammation-related proteins, as well as the number of regulated proteins connected to the cytoskeleton and motility.

**Figure 4:**
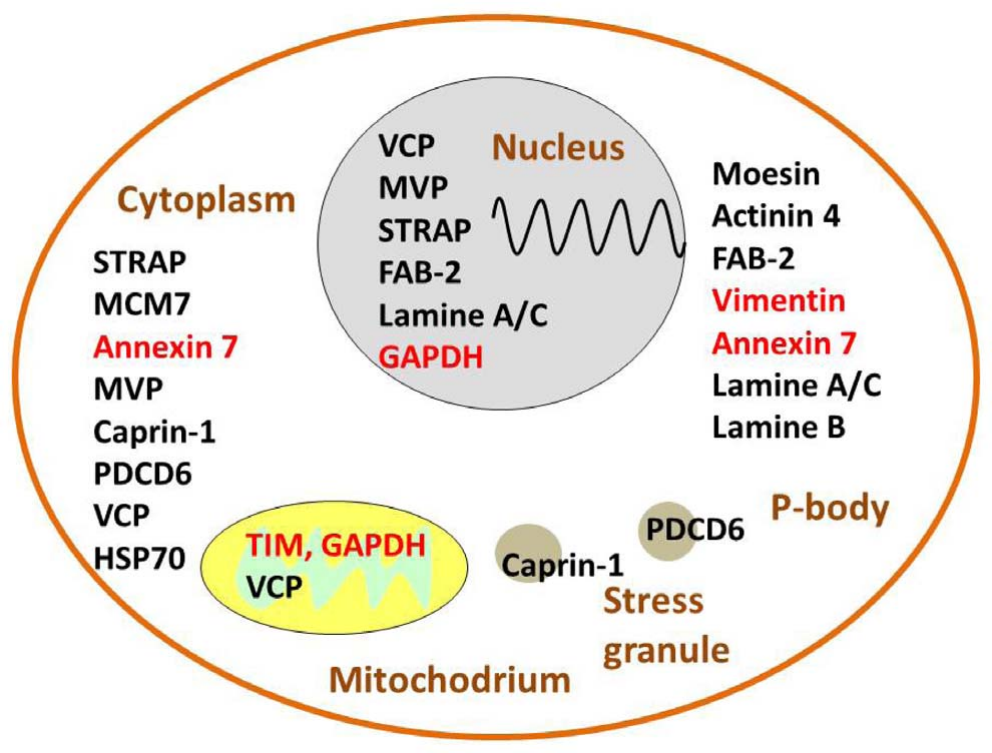
A diagram showing all proteins regulated in proton-irradiated BLM cells. The level of 17 proteins changed (>1.5×) in comparison with control. Thirteen proteins were upregulated (in black) and 4 were downregulated (in red). Here they are presented in four, not exclusive, groups: i) DNA repair and stress, ii) proliferation and survival control, iii) metabolic and iv) connected to motility and the cytoskeleton. ACTN 4 - α Actinin 4, Caprin-1 - Cytoplasmic activation/proliferation-associated protein-1, FAB-2 - Far upstream element binding protein 2, G3BP1 - RasGAP SH3-domain-binding protein 1, GADPH - Glyceraldehyde 3-phosphate dehydrogenase, MCM-7– Minichromosome Maintenance Protein 7, Moesin - Actin-regulatory protein, MVP - Major Vault Protein, PDCD6 - Programmed cell death 6, or apoptosis-linked gene-2, STRAP - Serine-threonine kinase receptor-associated protein, TIM - Triosephosphate isomerase, VCP – Transitional endoplasmic reticulum ATPase.

A closer look at the functions of proteins’ regulation after proton beam irradiation suggests that it is possible to classify them, simply for streamlining purposes, into four groups: i) DNA repair and stress, ii) proliferation and survival control, iii) metabolism and iv) connected to motility and the cytoskeleton (Fig. 4). This is an arbitrary classification, as some proteins have multiple functions and can play a role in more than one group.

Allowing cells to undergo several passages in culture to repair proton irradiation damage before carrying out the proteomic analysis ensures that the effects seen are long-term. Even a low dose of 3 Gy results in proteomic changes in the next generations of cells.

### Inducing DNA Repair and Stress Response

As expected after any kind of radiation treatment, proton beam irradiation upregulated protein levels engaged in DNA repair, such as MVP, Lamine A/C and Glyceraldehyde 3-phosphate dehydrogenase (GADPH). The last one participates in DNA repair via APE1 endonuclease [37]. What is more, the level of Transitional endoplasmic reticulum ATPase (VCP), known to be involved in chromatin associated degradation, was also increased. These changes are consistent with DNA repair after radiation.

The primary known function of **MVP (Major Vault Protein)** is multidrug resistance, but recent studies have demonstrated that it is also engaged in transport mechanisms, signal transmission, and the immune response. MVP plays a role in cytoplasmatic signal transduction cascades, and is involved in signal transduction through the nucleocytoplasmic transport of PTEN (nuclear phosphatase and tensin homologue deleted on chromosome 10). Nuclear PTEN plays a role in chromosome stability (reducing spontaneous double strand breaks), DNA repair, cell cycle arrest and cellular stability. MVP is engaged in the DNA repair mechanism (nonhomologous end joining) by interaction with Ku70/80. The level of MVP increased in response to various DNA damaging agents including irradiation [38]. MVP is recognized as a prognostic biomarker of radiotherapy resistance [39].

**Lamine A/C** A-type lamins control the transcription and degradation of proteins, play key roles in cell cycle regulation and DNA double-strand break repair. Importantly, the proteins regulated by A-type lamins– Rb family members, 53BP1 (Tumor suppressor p53-binding protein 1), BRCA1(breast cancer type 1 susceptibility protein) and RAD51– exert tumor suppressor functions, with their loss being associated with cancer susceptibility [40]. High levels of lamin A/C are followed by stimulation of cell growth, migration and invasion [41].

**VCP (Transitional endoplasmic reticulum ATPase, or p97 or Cdc48)** is highly abundant, accounting for about 1% of total cellular protein. Conserved hexameric p97 belongs to the type II AAA+ (ATPases associated with diverse cellular activities) family [42]. VCP is postulated to be an ubiquitin-selective chaperone, whose key function is to disassemble protein complexes. VCP is involved in a wide variety of cellular activities e.g., cell cycle, apoptosis, gene transcription, proteostasis, and DNA damage response autophagy and the regulation of chromatine. In response to UV, phosphorylated VCP modulates the function of RNA polymerase [43].

Several proteins related to mRNA regulation increased after proton beam irradiation. Caprin-1 (Cytoplasmic activation/proliferation-associated protein-1), involved in stress granules, and programmed cell death 6 (PDCD6) engaged in P-bodies were upregulated. Both constitutive P-bodies and stress-induced stress granules (SGs) are important for mRNA regulation upon stress, inhibiting protein synthesis to conserve energy for the repair of molecular damage. They contain mRNA and numerous proteins, and their content is highly dynamic. SGs also are known to modulate signaling balancing apoptosis and cell survival [44], [45]. Interestingly, our results show the upregulation of G3BP1 and/or HSP70 (Heat shock protein 70 kDa). Both these proteins are tightly connected to stress granules, as HSP70 is directly correlated with SG formation, and G3BP1 (RasGAP SH3-domain-binding protein 1) is a protein regulating mRNA stability and stress granule formation [44]. Furthermore, MVP and VCP are connected to transcription regulation, and Serine-threonine kinase receptor-associated protein (STRAP) and Far upstream element binding protein 2 (FAB-2) are associated with mRNA regulation.

**Cytoplasmic activation/proliferation-associated protein-1 (Caprin-1)** is a cytoplasmic phosphoprotein. Caprin-1 binds G3BP-1 (RasGAP SH3 domain binding protein-1), in cytoplasmic stress granules [46], formed in response to stress like heat, arsenite, unfolded protein response or toxins. G3BP-1 is marker for SGs, which stabilize specific mRNAs, control their translation and degradation and also control apoptosis by sequestering and nullifying apoptosis-promoting factors [47]. Overexpression of Caprin-1 induced the formation of cytoplasmic stress granules. Caprin-1 directly and selectively binds mRNA for c-Myc and cyclin D2 [46].

#### PDCD6 (programmed cell death protein 6, or apoptosis-linked gene-2, ALG-2)

PDCP6 interacts with PATL1, a component of the P-body, which is a cytoplasmic non-membranous granule composed of translation-inactive mRNAs and proteins involved in mRNA decay. PDCP6 co-localizes also with mRNA-decapping enzyme 1A, a marker of P-bodies [48]. PDCD6 in complex with Alix (ALG-2 (apoptosis-linked gene 2)-interacting protein X, known also as AIP1 or p95) induces calcium-dependent apoptosis and reduces tumorigenicity [49].

**STRAP** (**Serine-threonine kinase receptor-associated protein)** plays a role in mRNA splicing and cap-independent translation [50].

**FAB-2/FBP-2** (**Far upstream element binding protein 2 (FBP-2, KHSRP)** belongs to the FBP family, engaged in transcription mechanisms i.e. splicing, mRNA stabilization, and degradation. FBPs are special transcription factors; they recognize the ssFUSE, single strand DNA *cis* element located upstream of the promoter of a target gene (e.g c-myc) and bind torsionally stressed pre-existing single strand regions and up-regulate gene expression. FBP2 can promote the decay of labile mRNA machinery or favor the maturation of a select group of microRNA precursors. FBP2 serves as a component of both Drosha and Dicer complexes and regulates the biogenesis of a subset of miRNAs [51], [52].

### Proliferation and Survival Control

The largest group of proteins affected by proton beam irradiation in BLM cells is associated with the proliferation and pro-survival response, including STRAP, MCM-7, Annexin 7, MVP, Caprin-1, PDCD6, and VCP. However, it is not clear whether the overall effect is pro-survival or pro-apoptotic.

**STRAP** (**Serine-threonine kinase receptor-associated protein**), which regulates both transforming growth factor beta (TGF-β) and p53 signaling [53], is localized in both the cytoplasm and nucleus. On the one hand STRAP as regulator of Phosphoinositide-dependent kinase-1 and Apoptosis signal-regulating kinase 1, stimulates cell growth and can contribute to tumor progression by blocking TGF-β-mediated signaling, but on the other hand it potentiates p53-induced apoptosis through direct interaction with p53. These findings suggest that STRAP might have an ambivalent role in the regulation of cell growth [53]. However, the mechanism of STRAP regulation is not completely understood [50]. STRAP also physically interacts with B-MYB. *B*-*myb* is a member of the *Myb* family of transcription factors, which is ubiquitously expressed and involved in controlling cell proliferation and differentiation [53].

**MCM-7** (**Minichromosome Maintenance Protein 7)** is a member of the group of protein complexes that are essential for DNA replication licensing and the control of cell cycle progression from the G1 to the S phase. MCM belongs to the family of AAA+ proteins. MCM7 overexpression and amplification occurs in several human malignancies [54], [55], although MCM7 takes part in both oncogenic and tumor suppressor signaling pathways. MCM7 binds Rb, p107, p130 proteins, which exert control over the entry into the S phase of DNA replication and cellular proliferation [56]. MCM7 also binds androgen receptor (AR) which regulates cell growth and proliferation. MCM7 serves as a co-transcriptional and co-translational enhancing factor of AR [57]. A unique feature of the MCM7 genome is that it contains an intronic cluster of miRNA in intron 13. These miRNAs shut down the expression of multiple tumor suppressor genes like p21, transcription factor E2F1, Bcl-2-like protein 11, PTEN [58]. PTEN deficiency causes Akt (Protein Kinase B, or PKB) hyperactivition and in consequence tumor initiation and progression. MCM7 overexpreession fosters tumorigenesis in combination with the action of miRNAs [59]. MCM7 is also involved in the activation of the ATR-dependent S-phase checkpoint by agents that induce DNA replication stress e.g. UV or X radiation [60].

**Annexin A7 (ANXA7 or synexin)** is a ubiquitously expressed member of the multifunctional Ca/phospholipid-binding annexin family. It shows Ca^2+^ dependent GTPase activity and is involved in membrane fusion and exocytosis. Annexin A7 is a tumor suppressor in human prostate and breast cancers. It overcomes pathologic androgen-receptor (AR) dependent proliferation via retinoblastoma protein (Rb1/p105) [61]. On the other hand Annexin A7 correlates with tumor malignancy and lymph node metastasis. High levels of Annexin A7 in a tumor correlate strongly with poor survival of patients. Repression of Annexin A7 results in increased levels of cell apoptosis, down-regulated cell proliferation, inhibited cell motility ability, and decreased cell invasive capacity [62].

#### Major vault protein, MVP

Recent studies have shown that MVP can cooperate with Insulin-like Growth Factor 1 (IGF-1R) in preventing apoptosis by upregulation of B-cell lymphoma 2 family of proteins (Bcl-2) and downregulation of Bax. Tumor progression and resistance to chemotherapy and radiotherapy may also be activated through the suppression of Bax and upregulation of IGF-1R, resulting in increased proliferation and reduced apoptosis caused by upregulation of Bcl-2 and overexpression of altered p53 [63]. It was also shown that MVP binds to **COP1** (constitutively photomorphogenic 1 ubiquitin ligase). Mammalian COP1 causes prooncogene c-Jun proteasome-mediated degradation but COP1 can also act as a monomeric E3 ubiquitin ligase to directly ubiquitinate tumor suppressor p53. Under unstressed conditions MVP enters into direct physical contact with COP1 and suppress c-Jun-mediated transcription of activator protein 1 (AP-1) pathway. After UV irradiation, MVP becomes phosphorylated, releases COP1, and does not inhibit the AP-1 transcription anymore [64].

**Cytoplasmic activation/proliferation-associated protein-1 (Caprin-1)** level correlates with cellular proliferation [65], [66]. Suppression of the expression of human Caprin-1 resulted in a slowing of the proliferation rate, due to a prolongation of the G1 phase of the cell cycle [65]. The Caprin-1 gene is suppressed in response to the overexpression of the p53 gene, which suggests that this gene could be an important mediator of p53-dependent tumor growth suppression [66].

**PDCD6 (programmed cell death 6, or apoptosis-linked gene-2, ALG-2)** is a calcium-binding protein involved in cell proliferation and death. PDCD6 can induce calcium-dependent apoptosis and reduce tumorigenicity [67]. PDCD6 is ubiquitously localized in the cytoplasm; however, PDCD6 is translocated to the nucleus in response to DNA damage. PDCD6 is involved in the signaling cascade of the p53-responsive apoptotic machinery [68]. PDCD6 also plays a significant role in modulating cellular angiogenesis and it can inhibit tumor growth via the suppression of tumor angiogenesis [69]. There are conflicting reports concerning the level of PDCD6 in tumors. PDCD6 expression is up-regulated in lung cancer patients, while reduced PDCD6 expression is observed in gastric cancer, ovarian cancer tissues and cancer cell lines. Moreover, recent studies indicate that PDCD6 has a synergic pro-apoptotic effect with anti-cancer drugs through the activation of NF-κB pathway [70].

**VCP (Transitional endoplasmic reticulum ATPase, or p97 or Cdc48)** is a substrate for protein tyrosine phosphatase (PTPL1), which is important in cellular transformations [71]. PTPL1 can have either a pro-apoptotic effect (via the death receptor, Fas) or an anti-apoptotic impact (via Proto-oncogene tyrosine-protein kinase Src, Insulin-like growth factor 1 receptor, Human Epidermal Growth Factor Receptor 2 kinases) depending on the cellular context [43]. Elevated expression of VCP is observed in certain types of cancer tissues, e.g., colorectal carcinomas, pancreatic endocrine neoplasms, follicular thyroid cancer, hepatocellular and lung carcinoma [72], [73]. VCP increases the protection of cells against apoptotic stimuli through activation of the NFκB and Akt signaling pathways [74].

**G3BP1** **(RasGAP SH3-domain-binding protein 1**) or **HSP70** have been observed, although it is not clear which one gives the signal. G3BP1 is a protein regulating mRNA stability and stress granule formation and HSP70 is often induced in stress. Both proteins are connected to the cellular stress response. As mentioned above, Caprin-1 binds G3BP-1 stress granules [46].

### Downregulation of Glycolysis

We observed the downregulation of two glycolytic enzymes as a result of proton beam irradiation, namely TIM and GADPH. TIM and GADPH are enzymes catalyzing two consecutive reactions of glycolysis, step 5 (conversion of dihydroxyacetone phosphate to glyceraldehyde 3-phosphate by TIM) and step 6 (simultaneous phosphorylation and oxidation of glyceraldehyde-3-phosphate to 1,3-biphosphoglycerate). GADPH is also involved in membrane transport, tubulin bundling and cytoskeletal dynamics, stem cell regulation and DNA repair. The downregulation of two consecutive steps in glycolysis might lead to a switch to mitochondrial oxidation for energy production and a less oncogenic fenotype [75].

**TIM (Triosephosphate isomerase)** is a glycolytic enzyme which catalyses the conversion of dihydroxyacetone phosphate to glyceraldehyde 3-phosphate. Different isoformes of the protein were found in melanoma [76]. In our study TIM was identified as a phosphorylated form (at serine 58). Earlier proteomic studies revealed TIM upregulation (1.6 fold change) in melanoma patients [77].

**GADPH (Glyceraldehyde 3-phosphate dehydrogenase)** is a glycolytic enzyme tightly regulated at both transcriptional and posttranslational levels. GAPDH specifically catalyzes the simultaneous phosphorylation and oxidation of glyceraldehyde-3-phosphate to 1,3-biphosphoglycerate. GAPDH is also involved in membrane transport, tubulin bundling and cytoskeletal dynamics. GAPDH interacts with tRNA and a transcription factor Oct-1 implicated in stress responses, metabolic control, and poised transcription states, regulates normal and pathological stem cell function. Many factors such as calcium, insulin or hypoxia up-regulate GAPDH expression. The level of GAPDH is elevated in many cancers [78].

### Paradoxical Changes in Motility and Cytoskeleton

In metastatic melanoma cells, many proteins connected to cell motility, adhesion and migration are upregulated. For example, matrix metalloproteinase-2 and integrin αvβ3 are implicated in melanoma metastases and progression [79]. The BLM cell line, used in this study, is a metastatic melanoma, widely studied for its high invasive capabilities [28], [79], [80]. For example, the chemokine receptors CXCR3 and CXCR4 were shown to be expressed in the BLM cell line [80].

Proton ion irradiation decreased cell migration and invasion in a dose-dependent manner, strongly inhibited matrix metaloproteinase-2 activity in highly aggressive HT1080 human fibrosarcoma cells in vitro, and significantly decreased pulmonary metastasis in mouse osteosarcoma *in vivo* [81].

In the present work, several proteins known to influence cellular motility were affected after proton beam irradiation. Most distinctly, Vimentin was strongly downregulated, along with the downregulation of Annexin 7, whereas Moesin, Lamine A/C, Lamine B and α Actinin 4 were upregulated.

**Moesin (Actin-regulatory protein moesin)** belongs to the ezrin/radixin/moesin (ERM) family which provides a regulated linkage between the plasma membrane and the actin cytoskeleton. Moesin plays an important role in cell motility by connecting the actin cytoskeleton to a variety of membrane anchoring proteins. Moesin is an essential mediator for actin cytoskeleton remodeling [82]. Moesin controls the adhesion dependent activation of Rho and subsequent myosin II contractility during 3D collagen invasion by melanoma cells [83]. Increased moesin expression also promotes the epithelial–mesenchymal transition (EMT) by regulating adhesion and contractile elements for changes in actin filament organization [84].

**ACTN4 (α Actinin 4)** is an actin-binding protein associated with cell motility and metastasis in breast, colorectal, pancreatic, hepatocellular carcinoma, and ovarian cancers. ACTN4 crosslinks F-actin filaments into bundles to form filopodia. Overexpression of ACTN4 increases the migratory potential of cells [85].

#### FAB-2/FBP-2 (Far upstream element binding protein 2 (FBP-2, KHSRP)

FBPs are co-regulated with the microtubule destabilizer, cytosolic phosphoprotein stathmin (known as oncoprotein 18). FBP-2 primarily supports migration in different hepatocellular carcinoma cells [86].

**Lamins A/C and B** type are the main constituents of a polymeric network known as a nuclear lamina which plays a key role in the maintenance of genome localization, structure and function. Overexpression of Lamin A/C results in the stimulation of cell growth, migration and invasion. This oncogenic behaviour of lamin A/C is regulated through modulation of the PI3K/AKT/PTEN signaling pathway [41]. Lamin A regulates actin dynamics, and its overexpression leads to a loss of cell adhesion. This in turn increases cell motility and consequently increases the invasive potential of the tumor [87]. A-type lamins also influence the activity of oncogene β-catenin (via the interaction of the membrane protein emerin with the Wnt/β-catenin pathway) which also points to a possible role of A-type lamins in tumor progression [88]. In turn, B lamin deficiency is involved in the development of prostate cancer. B-deficient microdomains correlate with prostate cancer cell line aggressiveness and increased cell motility [89].

#### Annexin A7 (ANXA7 or synexin)

Repression of Annexin A7 results in increased levels of cell apoptosis, down-regulated cell proliferation, inhibited cell motility ability, and decreased cell invasive capacity [62].

**Vimentin** is one of the most widely expressed mammalian intermediate filament proteins. It is a multifunctional protein, regulating several different physiological functions, such as the structural integrity of cells and tissues, adhesion and migration, signal transduction, apoptotic and immune defense and the regulation of genomic DNA [90]. In the majority of cancers vimentin is overexpressed and associated with a metastatic phenotype and a poor prognosis for the disease outcome. Vimentin is a marker, as well as a key factor in regulating EMT, a critical event in the induction of cell motility [91]. Recent studies have revealed that vimentin is not only a diagnostic marker but also a hematogenous metastasis clinical predictor for melanoma [92].

A decrease in vimentin may lead to diminished metastatic potential. For example, in B16 melanoma cells treated with cyclohexamide a decrease in vimentin mRNa was seen, together with a decrease in the formation of lung metastastes in mice. What is more, this effect was reversible [93]. Similarly, withaferin A caused the dose-dependent inhibition of lung metastases in a breast cancer model [94].

Post-translation modifications of vimentin, specifically phosphorylation, are especially important for vimentin functioning. Vimentin contains a highly complex phosphorylation pattern involving a multitude of kinase specific sites which are recognized by many kinases, including Rho kinase, protein kinase C (PKC), cGMP kinase, Yes kinase, Raf-1 kinase, PAK kinase, and Aurora B kinase. Phosphorylation enhances the disassembly of vimentin into nonfilamentous (monomeric, dimeric, and tetrameric) particles, shifting the equilibrium between polymeric and depolymerized vimentin. Dephosphorylated vimentin exerts its influence on motility, adhesion, cell signaling, and cell survival. Similarly, phosphorylated, disassembled vimentin has been demonstrated to enhance the recycling of integrins subjected to endocytosis to the plasma membrane during cell migration [90]. The results of a proteomic 2DE experiment on melanoma cells confirm the multiisofomic pattern of vimentin [76]. It was attempted in the present study to confirm the changes in the level of vimetin by means of Western Blot analysis (data not shown), although the results were not statistically significant. The antibody applied (Vimentin (R28) Antibody #3932, Cell Signaling Technology) recognizes a synthetic peptide of vimetin which includes arginine at position 28. This residue is surrounded by serine and threonine amino acids, which are very often modified (phosphorylated). Because of the multiplicity of possible vimentin post-translational modifications, which may alter the affinity of the antibody for a protein, the Western Blot analysis is hampered especially in terms of quantitative analysis.

A decrease in vimentin level, as well as in Annexin 7, might suggest cell motility impairment, and decreased cell invasive capacity [62]. However, other proteins involved in motility and the cytoskeleton were upregulated. It is difficult, therefore, to assess the overall effect on cellular motility and migration. These proteins may change as the response of highly motile cells to stress, as part of their prosurvival response.

### Signalling Pathways Affected by Proton Beam Irradiation

Disturbances in several signaling pathways, such as MAPK-ERK, RAS, AKT/PI3, G1/S Cyclin/Cyclin-dependent kinases, p53, B-RAF are characteristic of melanoma progression [95], [96]. These pathways are connected with the modulation of proliferation, survival, the cell cycle, and cell differentiation. It is not surprising that some of the proteins upregulated after proton beam irradiation are also involved in these signaling pathways, critical for survival and proliferation (Table 2).

**Table 2:** Signaling pathways involving proteins upregulated after sublethal proton beam irradiation.

Pathway	Regulated proteins (reference)
MAPK	MVP (Valenciano et al., 2012) [63]
AKT/PI3	MVP (Blanco-Aparicio et al., 2007) [103]
	PDCD6 (Rho et al., 2012) [69]
	VCP (Braun and Zischka, 2008) [74]
	Lamin A/C (Kong et al., 2012) [41]
PTEN	MVP (Lara et al., 2011) [104]
	MCM-7 (Luo et al., 2011) [58], (Poliseno et al., 2010) [59]
	Lamin A/C (Kong et al., 2012) [41]
p-53	MVP (Lloret et al., 2009) [105]
	STRAP (Seong et al., 2007) [106]
	PDCD6 (Suzuki et al., 2012) [68]
	Caprin-1 (Saffari et al., 2009) [66]
Cell cycle	MCM-7, G1/S phase (Mukherjee et al., 2009) [56]
	Caprin-1, G1/S phase (Wang et al., 2005) [65]
	Caprin-1, cyclin D2 (Solomon S. et al., 2007) [46]
TGF-β	STRAP (Seong et al., 2007) [106]
nFκB	VCP (Braun and Zischka, 2008) [74]
	PDCD6 (Park et al., 2012) [70]

TGFβ acts as a tumor-derived immunosuppressor, an inducer of tumor mitogens, a promoter of carcinoma invasion, and a trigger of prometastatic cytokine secretion [97]. Photon radiation causes the upregulation of TGFβ secretion into the microenvironment and can induce fibro- sis by promoting the excessive synthesis of matrix components [98]. STRAP inhibits TGF-β signalling by stabilizing the association between TGF-β receptors and Smad7 or Smad3 (proteins that transduce extracellular signals from transforming growth factor beta ligands to the nucleus) [53], [99], [100]. The interaction of MCM7 with Rb is essential for transforming the growth factor (TGF)-β-induced blockade of entry into the S phase [56].

The relatively low dose of 3 Gy used in our studies, the time allowed for cell repair after irradiation might be the reasons we did not see the induction of signaling pathways described by others after proton irradiation, such as the direct apoptosis induction pathways p38/JNK [26].

### Comparison with other Melanoma Studies

Proteomic analysis of uveal melanoma from patients with distant metastases has shown increased expression of vimentin and TIM, among other proteins [77], in comparison to patients without metastases. In metastases of uveal melanomas, several proteins were found to be upregulated: Colony stimulating factor 2, Ficolin precursor, Haptoglobin -2 precursor, Hemopexin precursor, α-1-antitrypsin precursor, α-1-antichymotrypsin precursor, Vitronectin precursor, and Down syndrome cell adhesion molecule [101]. However, none of them were found to be modulated in our experiments. Proteomic analysis of melanoma metastases in comparison to primary tumors has shown upregulation of several proteins, implicated as metastasis-related proteins, such as lactate dehydrogenase, heat shock protein 90 KDa, glucose transporter 1, Macrophage migration inhibitory factor, Protein DJ-1, pyruvate kinase isozyme 2 and prohibitin-2 [102]. Again, none of these proteins were modulated in our study. Only GADPH, elevated in metastasis, was downregulated after proton beam irradiation in our cell line. Another comparative proteome analysis of two genetically, very closely related, melanoma cell lines with low- and high-metastatic potentials identified 42 proteins with increased levels in highly metastatic melanoma cells, while 68 showed decreased levels [98]. Four of the proteins with increased levels in highly metastatic melanoma cells were also seen in our study: Vimentin and TIM levels decreased after proton beam treatment, and STRAP and Actinin 4 increased.

Future work should cover other cancer cell lines, such as uveal melanoma or prostate cancer, both of which respond well to proton beam therapy. Perhaps proteomic analysis should also be performed a shorter time after irradiation.

## Conclusions

Proteomic analysis of the BLM melanoma cell line irradiated with a low dose of 3 Gy of proton beam shows a significant (more than 1.5×change) upregulation of 13 proteins and downregulation of 4 proteins. These proteins might be roughly grouped into four categories by function: (i) DNA repair and RNA regulation (VCP, MVP, STRAP, FAB-2, Lamine A/C, GAPDH), (ii) cell survival and stress response (STRAP, MCM7, Annexin 7, MVP, Caprin-1, PDCD6, VCP, HSP70), (iii) cell metabolism (TIM, GAPDH, VCP), and (iv) cytoskeleton and motility (Moesin, Actinin 4, FAB-2, Vimentin, Annexin 7, Lamine A/C, Lamine B). Of particular interest is the substantial decrease (2.3×) in vimentin, a marker of EMT and of the metastatic properties of melanoma [92].
